# Morbidity and health seeking behavior among children and adolescents (0–19 years): a household survey assessment in Northwestern Tanzania

**DOI:** 10.1186/s12913-025-13173-y

**Published:** 2025-07-31

**Authors:** Sophia Kagoye, Jim Todd, Milly Marston, Mark Urassa, Eveline T. Konje, Ties Boerma

**Affiliations:** 1 Department of Sexual and Reproductive Health, National Institute for Medical Research, Mwanza Research Centre, Mwanza, Tanzania; 2 Department of Epidemiology and Biostatistics, Catholic University of Health and Allied Sciences, Mwanza, Tanzania; 3 Department of Population Health, London School of Hygiene and Tropical Medicine, London, UK; 4 Community Health Science, University of Manitoba, Winnipeg, MB Canada

**Keywords:** Health seeking behavior, Morbidity, Treatment seeking, 5–19, Recent illness, Birth history

## Abstract

**Background:**

Information on morbidity and health-seeking behavior beyond early childhood is crucial for planning evidence-based interventions. Currently, data is limited to children under five. This study introduces a method for estimating morbidity and health-seeking behavior in older children and adolescents (5–19 years) using women’s birth histories from a household survey in northwestern Tanzania, comparing it with data on children under five.

**Methods:**

We conducted a household survey among women 15–49 years as part of the Magu Health and Demographic Surveillance from October 2020 to November 2021, including 16,896 children aged 0–19 living with their mothers. The study outcomes were the prevalence of reported illness in the last four weeks and health-seeking behavior, defined as visiting a health facility for recent illness. Modified Poisson regression analysis was performed, accounting for mothers as clusters and adjusting for child and mother characteristics. We compared the prevalence of recent illness and health-seeking behavior among older children and adolescents (5–19 years) with children under five within the same population.

**Results:**

Morbidity presented as the prevalence of any illness decreased with age, from 26.1% in children under-five to 10.4% among adolescents aged 15–19. Health seeking behavior also decreased with age, from 48.2% in children under-five to nearly 30% among adolescents aged 15–19. Types of illnesses reported were similar across age groups, with Fever/Malaria accounting more than two-thirds, followed by respiratory tract illnesses. Higher illness prevalence was noted in rural areas for both age groups. Health seeking behavior was higher among mothers with secondary education and above for both children under-fives (APR:1.22;95% CI: 1.02, 1.47) and 5–19-year-olds (APR: 1.31; 95% CI:1.01, 1.70). Additionally, those with health insurance also reported higher health seeking behavior (APR: 1.38; 95% CI:1.07, 1.78), while lower for children in rural households (APR: 0.72; 95% CI:0.61, 0.83), for 5–19-year-olds.

**Conclusions:**

Our findings on morbidity and health-seeking behavior demonstrate the importance of extending health monitoring beyond early childhood. The inequalities identified point to gaps in programming and health service delivery that require attention.

**Supplementary Information:**

The online version contains supplementary material available at 10.1186/s12913-025-13173-y.

## Background

In 2022, an estimated 6.2 million children 0–19 years died worldwide, including 4.9 million children under-five years and 1.3 million older children and adolescents aged 5–19, with more than half of these deaths occurring in sub-Saharan Africa [1]. The majority of these deaths were due to preventable causes [2]. For instance, in Tanzania, the leading causes of death were pneumonia and malaria among children under-five years of age [3], malaria and diarrhoeal diseases at 5–14 years and HIV/TB and malaria at 15–19 years [4].

Population level data on childhood morbidity and treatment are primarily collected through household surveys. The main survey programs, notably the Demographic and Health Survey (DHS) and Multiple Indicator Cluster Surveys (MICS), include questions on the occurrence of selected symptoms associated with the leading causes of death (fever, cough and difficult breathing and diarrhoea) during the two weeks preceding the survey, followed by questions on treatment seeking behavior [5, 6]. These questions, however, are only asked for children under five years of age [6].

Population data on morbidity and health seeking behavior among older children and adolescents (5–19 years) are however lacking [7], even though such data are important for planning, implementation and monitoring of health service delivery beyond early childhood, including the identification of specific subgroups that would benefit most from targeted healthcare resources and interventions. Even clinical data on childhood morbidity and treatment are limited [7]. Routine reports from health facilities could provide information on childhood diseases but data quality tends to be poor. In addition, the facility reports typically categorize treatment visits into under-five or as five years and older, which includes adults, precluding the ability to assess morbidity in children 5–19 years [8].

In this study, we report the results from a new method to collect population data on morbidity and health seeking behavior extending to older children and adolescents, 5–19 years in existing survey instruments. In a household survey of all women 15–49 years, we added questions to the birth history for each child living with a mother on illness in the past 4 weeks preceding the survey and their health seeking behavior. We show that these additions resulted in valuable and plausible data prevalence of the recent illness and health seeking behavior patterns for older children and adolescents which should be considered for as a valuable addition to household surveys. We compare the morbidity patterns and health seeking behavior to a health facility among older children and adolescents (5–19 years) with younger children under-five.

## Methods

### Study setting

This study was a nested cross-sectional household survey conducted among all women of reproductive age (15–49 years) in Magu Health and Demographic Surveillance study (HDSS). Magu HDSS is located in Magu District, 20 km east of Mwanza city, in the northwestern part of Tanzania, has been operational since 1994 in a contiguous area of nine villages (4 semi-urban and 5 rural), with a population of 54,024 in 2022 [9]. Up to 2022, there were a total of 40 HDSS rounds that were conducted at an average of every eight-month intervals. Nearly half of the population in Magu HDSS were children under 20 years.

There are six government health facilities in Magu HDSS, including one government health centre in the main semi-urban trading centre and five government dispensaries in the rural villages. Almost all households in Magu HDSS have access to the health facilities within a 5 km distance. All government facilities provide maternal and child health services, including other outpatient and inpatient services for all age groups [10]. Further details regarding the Magu HDSS are highlighted elsewhere [9, 11].

### Data

A household survey nested within Magu HDSS was conducted between October 2020 to November 2021 among women of reproductive age (15–49 years) living in Magu HDSS at the time of the survey. The sampling frame for the survey was obtained from the 37th HDSS round conducted in 2020. All eligible women 15–49 years were interviewed in their own households with a standardized questionnaire including household and individual characteristics, birth history of all children born to the women alive or dead, outpatient and inpatient service utilization and expenditure for the women, family planning, antenatal and delivery care, vaccination and use of insecticide treated nets for children born in the 3 years prior the survey.

The survey instrument was adapted from selected modules of the standard Demographic and Health Survey (DHS) questionnaire, with additions to the birth history (Comparisons with TDHS questions are done in the Appendix Table 1 in Additional file 1). The following questions were added about health seeking behavior for all sick children under 20 years of age, living with the mother (full questionnaire in the Additional file 2):

- Whether or not a child (under 20 years) was sick in the last 4 weeks prior the survey.
- Type of illness leading to seeking care (open-ended with pre-coded response categories): these included fever/malaria, diarrhoea, respiratory tract illnesses (cough/difficult in breathing), others.
- Whether or not treatment was given at home.
- Whether or not a child (under 20 years) was taken to a health facility.
- Type of health facility: with a coding list of all local facilities.

The survey instrument was administered using an electronic tablet to women of reproductive age, the interviewers were trained for two weeks and conducted a pilot within the same population to improve their skills. Data processing was done concurrently with data collection to allow for the generation of weekly field data quality checks. The tables were discussed with the field supervisor who then followed up with the interviewers. Further details on the survey instrument and its administration are highlighted elsewhere [12].

### Study population and sample size

The survey response rate was 81% which included 8,665 women of reproductive age and 23,996 children. The survey implementation was interrupted for a period of 4 months from April 2021 to July 2021 due to the COVID-19 pandemic. For this study, we included all children and adolescents aged 0–19 years who were alive and living with the mother at the time of the survey. We excluded children in the birth history that were above 20 years old (3,280), deceased (1,576), with missing information on age (10) and missing information on who they were living with (2) and those not living with the mother (2,232). The total sample size used for the final analysis was 16,896 children aged 0–19 years (Appendix Fig. 1 in Additional file 1).

### Measures

Main outcomes of interest in this study were prevalence of reported illness in the last 4 weeks prior the survey, which was binary coded as (0/1; No/Yes) and health seeking behavior defined as a visit to a health facility among those reported illness in the last 4 weeks), also binary outcome coded (0/1; No/Yes). All outcomes were assessed among children under-fives and older children and adolescents (5–19-year-olds).

The determinants of a child being sick in the last 4 weeks prior the survey and health seeking behavior were grouped into child and mother characteristics and these were selected based on previous literature in sub-Saharan Africa [13–16]. Child characteristics included in the analysis were sex (male/female), area of residence (semi-urban/rural) and type of illness (fever or malaria, diarrhoea, respiratory tract illnesses, others). Other illnesses reported for children under-five included: headache, worm infestations, convulsions and urinary tract infections; for older children and adolescents (5–19-year-olds): unspecified headache, epilepsy, sickle cell disease, urinary tract infections, anemia, peptic ulcer disease and jaundice. Mother’s characteristics included age of the mother in years, mother’s education (no education/complete primary education/secondary education and above)– used as a proxy for socioeconomic status, and health insurance status (No/Yes).

### Statistical analysis

Data analysis was performed using Stata version 18.0 [17]. Descriptive statistics were summarized using frequency and proportions for categorical variables and median with interquartile range for the continuous variables. The association of explanatory variables with outcomes of interest (prevalence of reported illness in the last 4 weeks and health seeking behavior) were tested using a Chi-square (2) test.

A multivariable logistic regression with random effects was initially considered to determine factors associated with health seeking behavior, taking into account the hierarchical nature of the data. However, this approach was discarded due to the drawbacks of logistic regression of over-estimating odds ratios and 95% confidence intervals for common outcomes (prevalence greater than 10%) [18]. Since in our case the prevalence of health seeking behavior was well over 10%, classical logistic regression was not used instead other alternatives to classical logistic regression were considered.

We conducted modified Poisson regression analysis (Poisson regression with a robust error variance) [18, 19], accounting for clustering at the mothers’ level as children are nested within mothers. Health seeking behavior was adjusted for sex of the child, area of residence, type of illness, age of the mother, mother’s education and health insurance status of the mother for both children under-five and 5–19-year-olds. Crude and Adjusted Prevalence ratios with respective 95% confidence intervals were used to interpret the magnitude of association, *p*-value of < 0.05 was considered statistically significant. Final model selection was based on the lowest Akaike Information Criteria [20].

## Results

### Background characteristics of children and mothers

Among 16,896 children in the study, the majority were children under-five (5,711), followed by those aged 5–9 (4,973), 10–14 (3,802) and 15–19 years old (2,410). Among all age groups, the male/female distribution was 50/50 and majority resided in rural villages of the HDSS.

Median age of the mothers increased with an increase in the age of children from 28 (IQR: 24,34) among children under-five to 33 (IQR: 28, 39), 38 (33, 42) and 41 (38, 45) years among 5–9, 10-14- and 15–19-year-olds, respectively. Most children’s mothers had completed primary education (ranging from 56.5% among children under-five to more than 60% among older adolescents aged 15–19 years) but few had health insurance (below 10% in all age-groups) (Appendix Table 2 in Additional file 1).

#### Prevalence of reported illness in the last 4 weeks

**Fig. 1:**
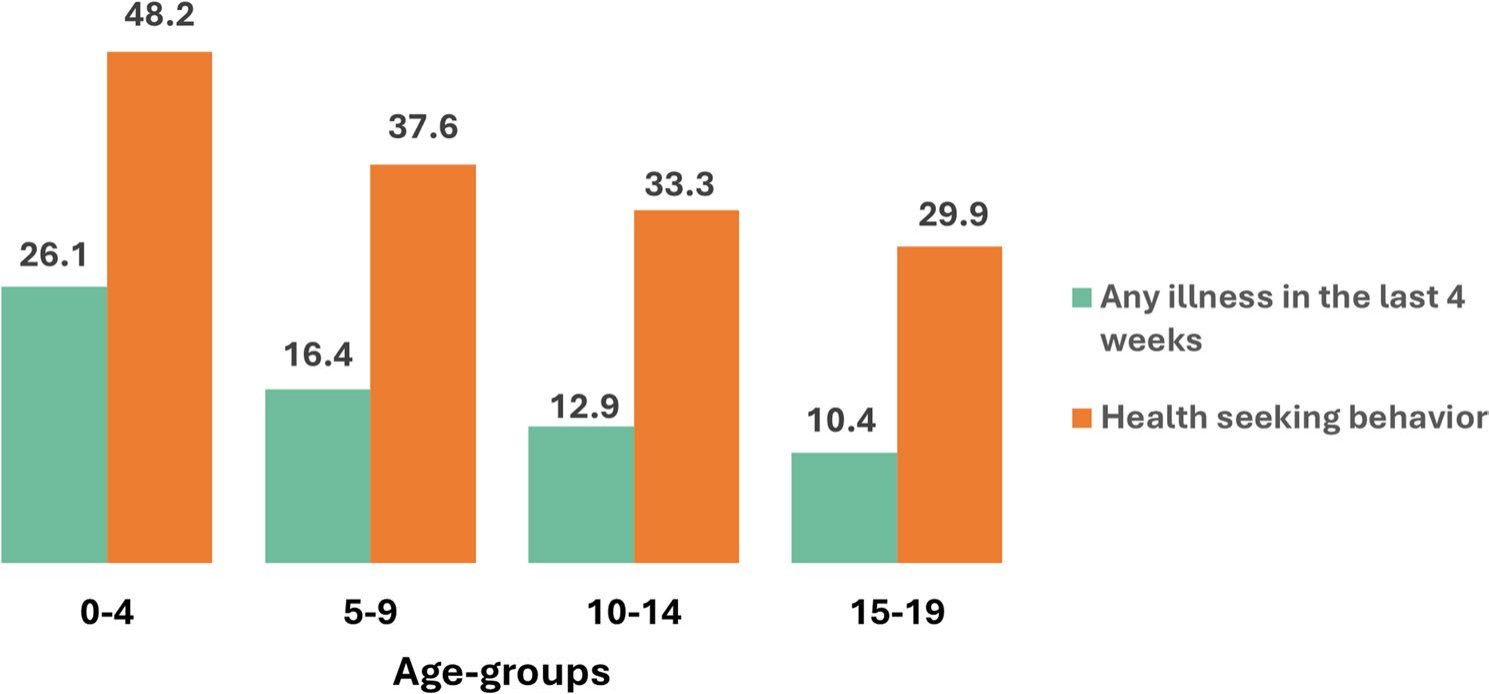
Prevalence of any illness in the last 4 weeks and health seeking behavior by age-groups

The prevalence of any illness in the last 4 weeks decreased with age from 26.1% among children under-five to 16.3% among 5–9-year-olds, 12.9% among 10–14-year-olds and 10.4% among 15–19-year-olds (Fig. 1 & Appendix Table 3 in Additional file 1).

**Fig. 2:**
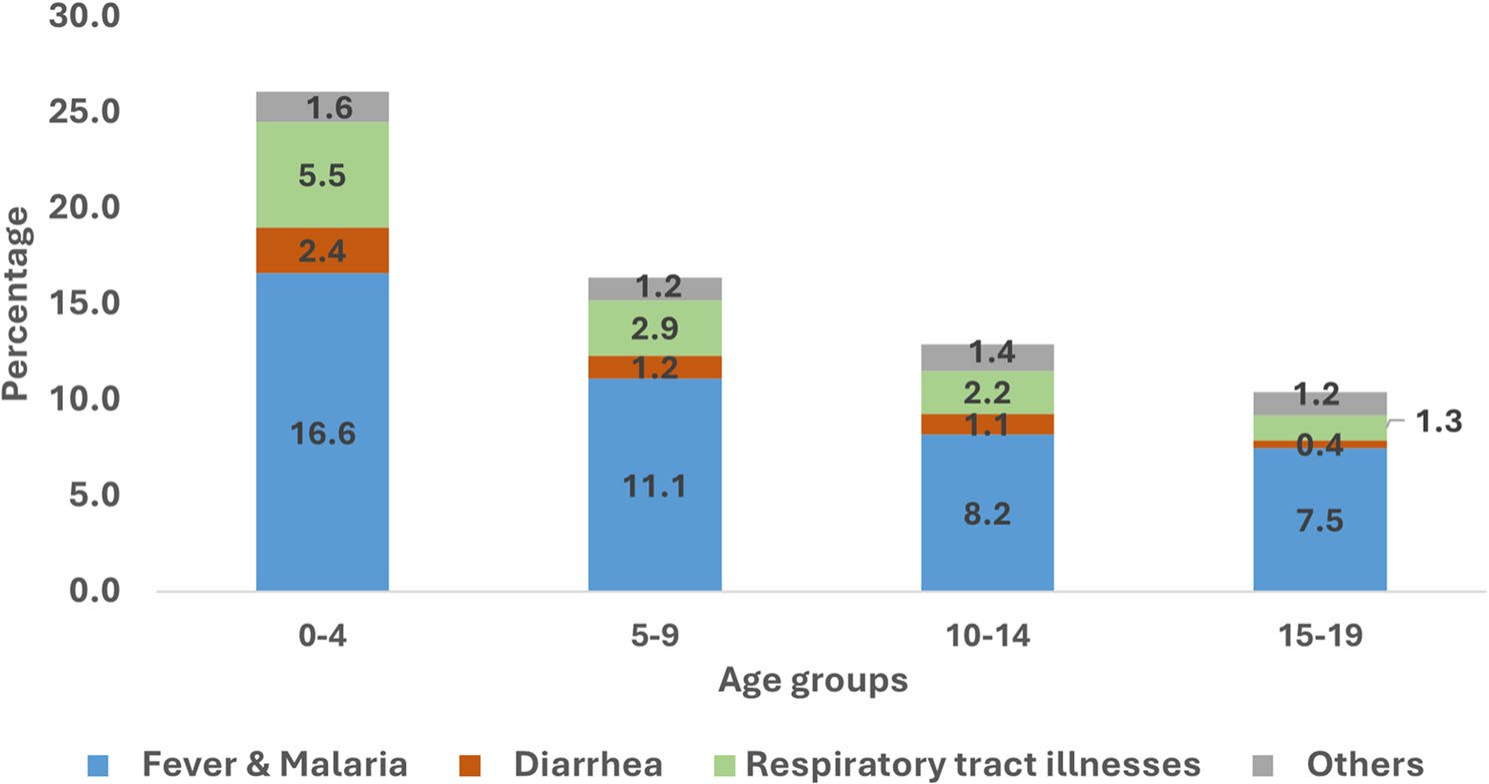
Types of illness reported in the past 4 weeks by age groups

The types of illnesses reported in the last 4 weeks did not vary much by age groups. Fever/malaria were common in all age groups, accounting for about two thirds of illnesses reported in all age groups and decreasing with age of the child. The prevalence of fever/malaria ranged from 16.6% among children under-five, to 11.1%, 8.2% and 7.5% among older children aged 5–9 years, and adolescents aged 10-14- and 15–19-year-old, respectively. Similarly, the relative importance of respiratory tract illnesses and diarrhea also decreased with age, ranging from 5.5% and 2.4% among children under-five to 1.3% and 0.4% among older adolescents aged 15–19-year-olds, for both respiratory tract illnesses and diarrhoea, respectively (Fig. 2 & Appendix Table 3 in Additional file 1). Multiple comorbidities were also reported across all age groups, with nearly 50% of respiratory tract illnesses, diarrhoea and other diseases co-existing with fever (Appendix Table 4).

#### Prevalence of any illness last 4 weeks by background characteristics

The prevalence of any illness in the last 4 weeks significantly varied by area of residence. The prevalence was higher in rural areas compared to semi-urban areas within the Magu HDSS among children under-five (28.2% compared to 23.0%) and 5–19-year-olds (16.1% compared to 11.2%). There were no statistically significant differences in how illness reported varied by other characteristics, for both children under-five and 5–19-year-olds (Table 1).

**Table 1:** Prevalence of any illness last 4 weeks by background characteristics

Sick in the past 4 weeks	0–4 (*N* = 5,711)		5–19 (*N* = 11,185)	
	1,491 (26.1)		1,556 (13.9)	
	*n* (%)	*p*-value	*n* (%)	*p*-value
Sex				
Male	734 (26.0)	*0.820*	778 (13.8)	*0.949*
Female	757 (26.2)		778 (14.0)	
Area of residence				
Semi-urban	525 (23.0)	*< 0.001*	555 (11.2)	*< 0.001*
Rural	966 (28.2)		1,001 (16.1)	
Mother characteristics				
Age of the mother				
15–24	366 (29.1)	*0.182*	10 (20.0)	*0.979*
25–34	473 (26.8)		81 (18.0)	
35+	244 (24.4)		219 (17.4)	
Mother’s education				
No education	318 (25.8)	*0.894*	465 (15.0)	*0.388*
Complete primary education	841 (26.1)		940 (13.6)	
Secondary and above	332 (26.6)		151 (13.2)	
Health insurance *				
No	1,425 (26.1)	*0.928*	1,486 (13.9)	*0.660*
Yes	66 (25.9)		70 (14.3)	

*p*-value shows comparison among the subgroups in each variable

#### Health seeking behavior

Health seeking behavior among those reported illness in the last 4 weeks, also varied by age groups, decreasing with age from 48.2% among younger children under-five to nearly 30% among 15–19-year-olds (Fig. 1). Across all age groups, nearly 50% were treated at home only, ranging from 48.6% among children under-five to one third (66.9%) among 15–19-year-olds (Appendix Table 3 in Additional file 1).

#### Health seeking behavior by background characteristics

Health seeking behavior significantly varied by type of illness and mother’s education level among children under-five, compared to variation by area of residence, type of illness, mother’s education level and health insurance among 5–19-year-olds but not by sex of the child in both age groups.

Children with respiratory tract illnesses were less likely to seek healthcare services. Health seeking behavior increased with mother’s education level for both children under-five and 5–19-year-olds. Among 5–19-year-olds reported sick in the last 4 weeks, health seeking behavior was higher in semi-urban areas compared to rural areas (43.1% compared to 30.6%). Also, health seeking behavior was higher among those with health insurance (51.4%) compared to their counterparts (34.2%) (Table 2).

**Table 2:** Health seeking behavior by background characteristics

Characteristic	0–4 (*n* = 1,491)		5–19 (*n* = 1,556)	
	n (%)	*p* -value	n (%)	*p* -value
All	718 (48.2)		545 (35.0)	
Sex				
Male	354 (48.2)	*0.956*	265 (34.1)	*0.425*
Female	364 (48.1)		280 (36.0)	
Area of residence				
Semi-urban	267 (50.9)	*0.124*	239 (43.1)	*< 0.001*
Rural	451 (46.7)		306 (30.6)	
Type of illness				
Fever/Malaria	486 (51.2)	*< 0.001*	390 (37.3)	*< 0.001*
Diarrhoea	77 (55.4)		33 (29.5)	
Respiratory tract illnesses	96 (30.8)		51 (19.8)	
Other	58 (64.4)		71 (50.0)	
Age of the mother (years)				
15–24	186 (50.8)	*0.063*	4 (40.0)	*0.901*
25–34	236 (49.9)		33 (40.7)	
35+	102 (41.8)		83 (37.9)	
Mother’s education				
No education	133 (41.8)	*0.007*	138 (29.7)	*< 0.001*
Complete primary education	405 (48.2)		333 (35.4)	
Secondary and above	180 (54.2)		74 (49.0)	
Health insurance				
No	686 (48.1)	*0.956*	509 (34.2)	*0.003*
Yes	32 (48.5)		36 (51.4)	

After adjusting for child and mother characteristics, among children under-five, health seeking behavior was 41% lower among those with respiratory tract illnesses compared to fever/malaria illness (APR: 0.59; 95% CI: 0.49, 0.71). Health seeking behavior increased by 22% among mothers with secondary education and above (APR: 1.22; 95% CI: 1.02, 1.47) (Table 3).

Among 5–19-year-olds, the prevalence of health seeking behavior was significantly lower by 28% among those residing in rural areas compared to semi-urban areas (APR: 0.72; 95% CI: 0.61, 0.83). The prevalence ratio was also lower among those with respiratory tract illnesses compared to fever/malaria illness (APR: 0.52; 95% CI: 0.40, 0.68). Similar to younger children under-five, the prevalence ratio of health seeking behavior also increased with mother’s education level, by 10% and 31% among those with complete primary education (APR: 1.10; 0.91, 1.32) and secondary education and above (APR: 1.31; 95% CI: 1.01, 1.70), respectively. The prevalence of health seeking behavior was also higher among those with health insurance by 38% compared to their counterparts (APR: 1.38; 955 CI: 1.07, 1.78) (Table 3).

**Table 3:** Determinants of health seeking behavior

Characteristic	0–4 (*n* = 1,491)		5–19 (*n* = 1,556)	
	Crude analysis	Adjusted analysis	Crude analysis	Adjusted analysis
	CPR (95% CI)	APR (95% CI)	CPR (95% CI)	APR (95% CI)
Sex				
Male	1	1	1	1
Female	1.00 (0.90, 1.11)	1.01 (0.91, 1.12)	1.06 (0.92, 1.21)	1.04 (0.91, 1.19)
Area of residence				
Semi-urban	1	1	1	1
Rural	0.92 (0.82, 1.03)	0.92 (0.82, 1.04)	0.71 (0.61, 0.83) ***	0.72 (0.61, 0.83) ***
Type of illness				
Fever & Malaria	1	1	1	1
Diarrhea	1.08 (0.92, 1.27)	1.09 (0.92, 1.28)	0.78 (0.58, 1.07)	0.81 (0.60, 1.11)
Respiratory tract illnesses	0.60 (0.50, 0.73) ***	0.59 (0.49, 0.71) ***	0.53 (0.41, 0.70) ***	0.52 (0.40, 0.68) ***
Other	1.26 (1.07, 1.48) **	1.26 (1.07, 1.49) **	1.34 (1.00, 1.63) **	1.42 (1.07, 1.71) ***
Age of the mother (years)	0.99 (0.98, 0.99) *	0.99 (0.98,0.99) *	0.98 (0.97, 0.99) **	0.98 (0.97, 0.99) **
Mother’s education				
No education	1	1	1	1
Complete primary education	1.15 (0.98, 1.35)	1.13 (0.96, 1.32)	1.19 (0.98, 1.45)	1.10 (0.90, 1.31)
Secondary and above	1.30 (1.08, 1.55) **	1.22 (1.02, 1.47) *	1.65 (1.29, 2.11) ***	1.31 (1.01, 1.70) *
Health insurance				
No	1	1	1	1
Yes	1.01 (0.77, 1.32)	0.97 (0.74, 1.27)	1.50 (1.16, 1.95) **	1.38 (1.07, 1.78) *

*CPR* Crude Prevalence ratio, *APR*Adjusted Prevalence ratio,*CI* Confidence interval

*p< 0.05,**p< 0.01,***p< 0.001

## Discussion

We reported on morbidity patterns and health seeking behavior to a health facility among older children and adolescents (5–19 years) compared to younger children under-five at a subnational level in Tanzania. To reduce the information gap on recent illness and morbidity among older children and adolescents (5–19 years), we extended data on recent illness and health seeking behavior to children and adolescents living with their mothers through questions added to the birth history in a household survey of women 15–49 years. Our method yielded plausible results on the prevalence of recent illness and health seeking behavior, including age patterns and disparities.

The prevalence of any illness for the last 4 weeks decreased with age, from 26.1% among children under five to 10% among older adolescents (15–19 years). Health-seeking behavior also declined with age, consistent with findings from studies conducted in Kenya and Malawi [14, 21]. A similar age pattern was also observed in mortality levels in the same area [22]. There is a possibility that as a child grows older, mothers are likely to perceive their ailments as less concerning compared to younger children, leading to lower health care seeking. Also, since recent illness and health seeking behavior in our study are based on maternal reports for children up to 19 years, we are likely to miss older adolescents who may self-manage their illness or those that do not communicate it to their mothers, this may result into under-reporting of recent illness and health seeking behavior among older adolescents in our study.

In both under-fives and 5–19 years, reported illness in the last 4 weeks was more common in rural areas compared to semi-urban areas. Similar observations were reported elsewhere for children under-five [14, 16, 23]. For older children and adolescents (5–19 years) in the Magu HDSS, we reported a higher risk of dying for children and adolescents residing in rural areas compared to semi-urban areas [22], although this was mostly driven by non-communicable diseases mortality [4]. In this study, recent illnesses due to diarrhoea, respiratory tract infections and other illnesses which also include non-communicable diseases were more common in rural children. This indicates that different strategies may be needed when it comes to address the urban/rural health in childhood. Health insurance coverage was low but did have a positive impact on service utilization. New ways to enhance uptake of (community) health insurance programs are needed [10].

Looking into the determinants of health seeking behavior, mother’s education was a significant determinant of health seeking behavior in both age groups, similar to what was observed in other studies in sub-Saharan Africa for children under-five [14, 23–25]. Educated mothers are more likely to access health information, be more knowledgeable of common childhood problems thus advocate for appropriate care seeking. This underscores the importance of education in improving child survival not just for younger children, however extending to older children and adolescents (5–19 years).

Overall, comparing morbidity and health-seeking behaviors across these age groups was crucial in identifying age-specific health risks, barriers to healthcare access, and different service needs. Such comparative insights are vital for designing targeted interventions, optimizing resource allocation, and developing strategies that effectively address the unique health challenges faced by each age group, thereby contributing to improved health outcomes for both children under five and adolescents.

Furthermore, to contextualize our findings, we benchmarked our study’s findings on under-five morbidity and health-seeking behavior with the national data from TDHS 2022, accounting for methodological differences between the two. We observed that illness prevalence was in the same ballpark after taking the different recall periods into account. Health-seeking behavior was lower, with 48% of caregivers in Magu HDSS seeking care compared to 64% in TDHS. This difference may be genuine or due to the longer recall period in our study. Evidence from other studies suggests that longer recall periods tend to underestimate both disease prevalence and healthcare utilization [26]. In addition, the use of open-ended questions in our survey as opposed to specific symptom questions in the DHS may contribute to these differences. Future research could focus on adding questions on specific signs and symptoms to the birth history for all children rather than open-ended questions. Some of the additional questions on type of treatment received could also be considered, although one would probably want to limit the number of questions as part of a birth history.

Our study was unable to assess validity of the new questions, which, however, is not any different from the DHS questions on morbidity and health seeking behavior. External comparisons were limited to the national DHS results for under-fives, as no data are available for children 5–19 years. While Magu HDSS children may have different morbidity and treatment patterns from other children in Tanzania, our previous work has shown that Magu HDSS is close to Tanzania’s averages for almost all indicators [9]. Another limitation is that we did not capture the effect of some other variables which have been shown to have an association with health seeking behavior, such as distance to a health facility, severity of illness and the qualitative context of health seeking behavior models [16, 27]. Also, with a longer recall period of four weeks compared to two weeks, we are more likely to underestimate the illness prevalence and health seeking behavior, as shown in other studies [26]. Finally, our study depended on reported information from the mothers on the child illnesses, some illnesses are likely to be omitted or other over-reported, and we were unable to estimate the extent of under or over-reporting of illnesses.

## Conclusions

Our study underscores the usefulness of the methods in determining recent illness and health seeking behavior among children beyond five years, as indicated by the plausibility of the results. The observed inequalities in morbidity and health-seeking behavior provide critical information for programs. Such data on morbidity and health seeking behavior collected by adding a few questions to the birth history in household surveys address an important void for children over the age of 5 years.

## Supplementary Information

Supplementary Material 1.

Supplementary Material 2.

## Data Availability

The datasets used and/or analysed during the current study are available from the corresponsing author on reasonable request.

## Abbreviations

Magu HDSS: Magu health and demographic surveillance study
MICS: Multiple indicator cluster surveys
TDHS: Tanzania demographic and health survey
