# Supplementary material for: Morbidity and health seeking behavior among children and adolescents (0–19 years): a household survey assessment in Northwestern Tanzania
Source: BMC Health Serv Res. 2025 Jul 31;25:1011. doi: 10.1186/s12913-025-13173-y (PMC12315430; doi:10.1186/s12913-025-13173-y)
Supplement: Supplementary file 1 — Supplementary Material 1. [file 12913_2025_13173_MOESM1_ESM.pdf]

Additional file 1

Appendix

Appendix Table 1: Differences between Demographic and Health Survey Questionnaire and Women Survey questionnaire for key modules used in the study (Recent child illness and treatment seeking)

**Indicator**	**Demographic and Health Survey Questionnaire**	**Women survey Questionnaire**
Respondent	Mother	Mother
Reference period	2 weeks	4 weeks
Recent illness	For children that are alive, living with the mother, born 5 years prior the survey:<br>1. Has (NAME) had diarrhea in the last 2 weeks?<br>2. Has (NAME) been ill with a fever at any time in the last 2 weeks?<br>3. Has (NAME) had an illness with a cough at any time in the last 2 weeks?<br>4. Has (NAME) had fast, short, rapid breaths or difficulty breathing at any time in the last 2 weeks?	For all children that are alive, living with the mother<br>1. Has (NAME) been sick in the last 4 weeks?<br>2. What was the problem?
Treatment seeking	1. Did you seek advice or treatment from any source?<br>2. Where did you seek advice or treatment?	1. Did you give (NAME) any treatment at home?<br>2. Did you take him/her to a health facility?<br>3. IF YES: which health facility?

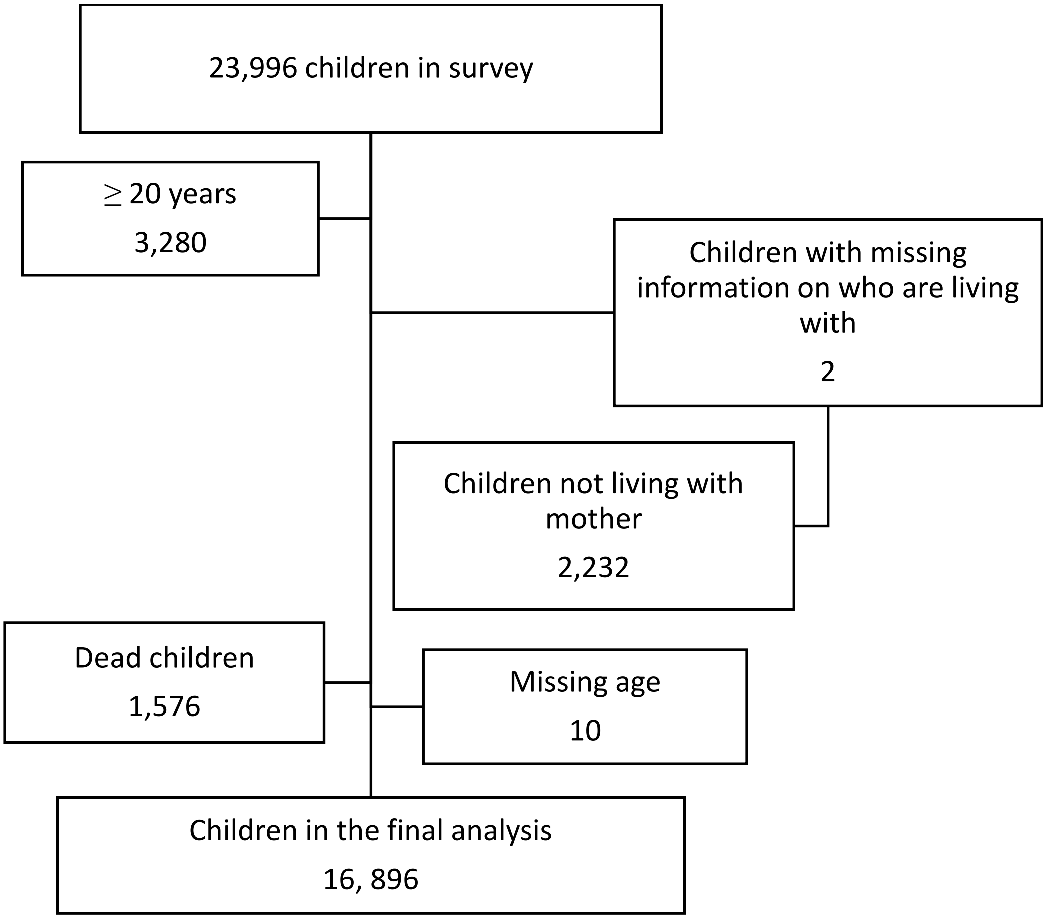

Appendix Figure 1: Flowchart for selection of study participants

Appendix Table 2: Background characteristics of children and mothers (Children N=16,896; Mothers N= 5,785)

	Child characteristics (N=16,896)			
	**0-4**	**5-9**	**10-14**	**15-19**
**Total N**	**5,711**	**4,973**	**3,802**	**2,410**
	**n (%)**	**n (%)**	**n (%)**	**n (%)**
**Sex**				
Male	2,825 (49.5)	2,495 (50.2)	1,930 (50.8)	1,199 (49.8)
Female	2,886 (50.5)	2,478 (49.8)	1,872 (49.2)	1,211 (50.2)
**Area of residence**				
Semi-urban	2,280 (39.9)	2,163 (43.5)	1,710 (45.0)	1,091 (45.3)
Rural	3,431 (60.1)	2,810 (56.5)	2,092 (55.0)	1,319 (54.7)
**Wealth tertiles***				
Poorest	1,636 (33.5)	1,930 (43.6)	1,161 (33.4)	762 (34.1)
Middle	1,642 (33.6)	1,037 (23.5)	1,157 (33.3)	734 (32.8)
Richest	1,606 (32.9)	1,457 (32.9)	1,158 (33.3)	741 (33.1)
**Mother characteristics**				
	**0-4**	**5-9**	**10-14**	**15-19**
Total N	**5,711**	**4,973**	**3,802**	**2,410**
	**n (%)**	**n (%)**	**n (%)**	**n (%)**
**Age of the mother**				
15-24	1,743 (30.5)	290 (5.8)	6 (0.2)	0 (0.0)
25-34	2,620 (45.9)	2,520 (50.7)	1,210 (31.8)	211 (8.8)
35+	1,348 (23.6)	2,163 (43.5)	2,586 (68.0)	2,199 (91.2)
Median (IQR)	*28 (24, 34)*	*33 (28, 39)*	*38 (33, 42)*	*41 (38, 45)*
**Mother's marital status**				
Never married	510 (8.9)	219 (4.4)	103 (2.7)	58 (2.4)
Married/Cohabiting	4,792 (83.9)	4,231 (85.1)	3,195 (84.0)	1,955 (81.1)
Separated/Divorced/Widowed	409 (7.2)	523 (10.5)	504 (13.3)	397 (16.5)
**Mother's education**				
No education	1,234 (21.6)	1,220 (24.5)	1,130 (29.7)	759 (31.5)
Complete primary education	3,227 (56.5)	3,006 (60.5)	2,394 (63.0)	1,531 (63.5)
Secondary and above	1,250 (21.9)	747 (15.0)	278 (7.3)	120 (5.0)
**Health insurance**				
No	5,453 (95.5)	4,742 (95.4)	3,640 (95.7)	2,302 (95.5)
Yes	255 (4.4)	226 (4.5)	158 (4.2)	106 (4.4)
Missing	3 (0.1)	5 (0.1)	4 (0.1)	2 (0.1)
**Wealth tertiles**				
Poorest 33%	1,636 (28.7)	1,930 (38.8)	1,161 (30.5)	762 (31.6)
Middle	1,642 (28.7)	1,037 (20.8)	1,157 (30.4)	734 (30.5)
Richest 33%	1,606 (28.1)	1,457 (29.3)	1,158 (30.5)	741 (30.7)
Missing	827 (14.5)	549 (11.1)	326 (8.6)	173 (7.2)

Appendix Table 3: Illness prevalence and health seeking behavior disaggregated by five-year age groups (0-19 years)

	Age groups			
	**0-4**	**5-9**	**10-14**	**15-19**
**Total N**	**5,711**	**4,973**	**3,802**	**2,410**
	**n (%)**	**n (%)**	**n (%)**	**n (%)**
**Prevalence of any illness, past 4 weeks**	1,491 (26.1)	813 (16.4)	492 (12.9)	251 (10.4)
**Prevalence by types of illness**				
Fever & Malaria	950 (16.6)	553 (11.1)	312 (8.2)	180 (7.5)
Diarrhea	139 (2.4)	58 (1.2)	44 (1.1)	10 (0.4)
Respiratory tract illnesses	312 (5.5)	143 (2.9)	83 (2.2)	31 (1.3)
Others	90 (1.6)	59 (1.2)	53 (1.4)	30 (1.2)
**Health seeking behavior**				
No	48 (3.2)	22 (2.7)	19 (3.9)	8 (3.2)
Home treatment only	725 (48.6)	485 (59.6)	309 (62.8)	168 (66.9)
Taken to a health facility*	718 (48.2)	306 (37.6)	164 (33.3)	75 (29.9)

*67% of them were also treated at home before being taken to the health facility

Appendix Table 4: Number of children showing cases with multiple conditions in major sub-groups, disaggregated by five-year age groups (0-19 years)

	Age groups			
	**0-4**	**5-9**	**10-14**	**15-19**
**Total N**	**5,711**	**4,973**	**3,802**	**2,410**
	**n**	**n**	**n**	**n**
**Number with any illness, past 4 weeks**	1,491	813	492	251
**Number by types of illness (including multiple conditions)**				
Fever & Malaria	950	553	312	180
Diarrhoea	55	35	29	10
Diarrhoea + Fever	52	16	12	0
Diarrhoea + Fever + Respiratory Infections + Others	32	7	3	0
Respiratory tract illnesses	183	74	34	16
Respiratory tract illnesses + Fever	125	63	46	12
Respiratory tract illnesses + Fever + Others	4	6	3	3
Others	61	40	38	25
Others + Fever	29	19	15	5

Appendix Table 5: Prevalence of any illness in the past 4 weeks by background characteristics stratified by five-year age groups (0-19 years)

	Age groups							
	0-4 (n=5,711)		5-9 (n=4,973)		10-14 (n=3,802)		15-19 (n=2,410)	
	n (%)	*p*-value	n (%)	*p*-value	n (%)	*p*-value	n (%)	*p*-value
**Sick in the past 4 weeks**	1,491 (26.1)		813 (16.4)		492 (12.9)		251 (10.4)	
**Sex**								
Male	734 (26.0)	*0.820*	407 (16.3)	*0.267*	245 (12.7)	*0.899*	126 (10.5)	*0.528*
Female	757 (26.2)		406 (16.4)		247 (13.2)		125 (10.3)	
**Area of residence**								
Semi-urban	525 (23.0)	*<0.001*	298 (13.8)	*<0.001*	164 (9.6)	*<0.001*	93 (8.5)	*0.002*
Rural	966 (28.2)		515 (18.3)		328 (15.7)		158 (12.0)	
**Wealth tertiles***								
Poorest	436 (26.6)	*0.208*	344 (17.8)	*0.092*	186 (16.0)	*0.009*	98 (12.9)	*0.119*
Middle	404 (24.6)		157 (15.1)		138 (11.9)		65 (8.9)	
Richest	411 (25.6)		232 (15.9)		137 (11.8)		73 (9.8)	
**Age of the mother**								
15-24	356 (28.3)	*0.374*	10 (20.0)	*0.678*	-	*0.902*	-	*0.700*
25-34	474 (26.8)		69 (18.2)		8 (13.8)		1 (7.7)	
35+	249 (24.9)		141 (19.4)		55 (14.4)		24 (15.8)	
**Mother's marital status**								
Never married	140 (27.4)	*0.069*	40 (18.3)	*0.425*	11 (10.7)	*0.421*	4 (6.9)	*0.573*
Married/Cohabiting	1,226 (25.6)		676 (16.0)		404 (12.6)		209 (10.7)	
Separated/Divorced/Widowed	125 (30.6)		97 (18.6)		77 (15.3)		38 (9.6)	
**Mother's education**								
No education	318 (25.8)	*0.894*	212 (17.4)	*0.837*	164 (14.5)	*0.047*	89 (11.7)	*0.319*
Complete primary	841 (26.1)		482 (16.0)		303 (12.7)		155 (10.1)	
education							7 (5.8)	
Secondary and above	332 (26.6)		119 (15.9)		2 (9.0)			
**Health insurance ***								
No	1,425 (26.1)	*0.928*	776 (16.4)	*0.867*	467 (12.8)	*0.505*	243 (10.6)	*0.515*
Yes	66 (25.9)		37 (16.4)		25 (15.8)		8 (7.6)	

Appendix Table 6: Comparison of Recent illness and health seeking behavior TDHS 2022 and Magu HDSS

Survey	Recall period	Recent illness (%)	Health seeking behavior (%)
TDHS 2022	2 weeks	16.8 (15.7, 18.1)	64.0 (60.5, 67.2)
Magu HDSS (Women survey)	4 weeks	26.1 (25.0, 27.3)	48.0 (45.6, 50.7)
